# Reduced Expression of the ROCK Inhibitor Rnd3 Is Associated with Increased Invasiveness and Metastatic Potential in Mesenchymal Tumor Cells

**DOI:** 10.1371/journal.pone.0014154

**Published:** 2010-11-30

**Authors:** Cristina Belgiovine, Roberta Frapolli, Katiuscia Bonezzi, Ilaria Chiodi, Francesco Favero, Maurizia Mello-Grand, Angelo P. Dei Tos, Elena Giulotto, Giulia Taraboletti, Maurizio D'Incalci, Chiara Mondello

**Affiliations:** 1 Istituto di Genetica Molecolare, Consiglio Nazionale delle Ricerche (CNR), Pavia, Italy; 2 Istituto di Ricerche Farmacologiche Mario Negri, Milan, Italy; 3 Istituto di Ricerche Farmacologiche Mario Negri, Bergamo, Italy; 4 Cancer Genomics Laboratory, Fondazione Edo ed Elvo Tempia, Biella, Italy; 5 Ospedale Generale di Treviso, Treviso, Italy; 6 Dipartimento di Genetica e Microbiologia, Università di Pavia, Pavia, Italy; University of Birmingham, United Kingdom

## Abstract

**Background:**

Mesenchymal and amoeboid movements are two important mechanisms adopted by cancer cells to invade the surrounding environment. Mesenchymal movement depends on extracellular matrix protease activity, amoeboid movement on the RhoA-dependent kinase ROCK. Cancer cells can switch from one mechanism to the other in response to different stimuli, limiting the efficacy of antimetastatic therapies.

**Methodology and Principal Findings:**

We investigated the acquisition and molecular regulation of the invasion capacity of neoplastically transformed human fibroblasts, which were able to induce sarcomas and metastases when injected into immunocompromised mice. We found that neoplastic transformation was associated with a change in cell morphology (from fibroblastic to polygonal), a reorganization of the actin cytoskeleton, a decrease in the expression of several matrix metalloproteases and increases in cell motility and invasiveness. In a three-dimensional environment, sarcomagenic cells showed a spherical morphology with cortical actin rings, suggesting a switch from mesenchymal to amoeboid movement. Accordingly, cell invasion decreased after treatment with the ROCK inhibitor Y27632, but not with the matrix protease inhibitor Ro 28-2653. The increased invasiveness of tumorigenic cells was associated with reduced expression of Rnd3 (also known as RhoE), a cellular inhibitor of ROCK. Indeed, ectopic Rnd3 expression reduced their invasive ability *in vitro* and their metastatic potential *in vivo*.

**Conclusions:**

These results indicate that, during neoplastic transformation, cells of mesenchymal origin can switch from a mesenchymal mode of movement to an amoeboid one. In addition, they point to Rnd3 as a possible regulator of mesenchymal tumor cell invasion and to ROCK as a potential therapeutic target for sarcomas.

## Introduction

Neoplastic transformation is a gradual process, during which cells acquire successive mutations, which mainly cause the loss of proliferation control, the ability to divide indefinitely and invade other tissues. A critical step in the development of a malignant cancer is tumor cells' acquisition of the capacity to migrate and invade tissues [1]. Different molecular mechanisms are responsible for the acquisition of a migratory and invasive phenotype, such as changes in signal transduction pathways involving tyrosine kinases, changes in cytoskeletal organization and in cell adhesion.

The small GTPases of the Rho family, mainly RhoA, cdc42 and Rac [2]–[4], play a pivotal role in regulating the actin cytoskeleton and cell movement. The RhoA/ROCK and Rac signalling pathways are required for respectively amoeboid and mesenchymal movements, which are the main types of movements adopted by tumor cells [5], [6]. The mesenchymal movement is typical of cells displaying an elongated morphology in a 3D environment; it requires integrin attachment to the extracellular matrix, formation of focal contacts and pericellular proteolysis. Besides cancer cells of mesenchymal origin, carcinoma cells can adopt this type of migration, after undergoing an epithelial-mesenchymal transition (EMT) [7].

In contrast, some carcinoma cells can move very fast with an amoeboid shape [8], [9]; *in vitro* studies in 3D environments have shown that this movement is typical of cells with a rounded morphology and is associated with the formation of actin cortical rings and membrane blebbings. The amoeboid movement, which is potentially faster than the mesenchymal one, does not rely on integrins, focal contacts and extracellular matrix degrading enzymes; it is mainly based on contraction of actomyosin filaments which enables the cells to squeeze through the extracellular matrix without the requirement of matrix degradation.

Acto-myosin contractility is strictly dependent on the activity of the RhoA-dependent kinase ROCK [9]–[11] and chemical blockade of ROCK inhibits amoeboid movement [10]. Cells can switch from one type of invasion mechanism to the other in response to changes in protein expression, or after treatment with certain compounds [5], [12]–[18]. For example, inhibition of matrix proteases or integrins inhibits mesenchymal migration and promotes a transition towards amoeboid movement [19], [20]. Identifying the factors and genes controlling the different types of motility could help direct therapeutic strategies aimed at reducing invasion.

Amoeboid migration has been recognized as an important mechanism of invasion and metastasis of carcinoma cells and, more recently, of sarcomas [21]. However, little is known about the possible association between sarcoma malignant progression and acquisition of the amoeboid phenotype. In this paper, we have exploited the cen3tel model of isogenic cells at different stages of transformation, from normal fibroblasts up to metastatic cells, to study changes in the migratory and invasive potential accompanying human fibroblast neoplastic transformation. The human fibroblast cell line cen3tel, obtained in our laboratory by telomerase immortalization, gradually underwent spontaneous neoplastic transformation [22]–[24]. Studying cells at different phases of transformation, we could show that an early event during transformation was the loss of expression of the *CDKN2A* locus, followed by inactivation of p53 and overexpression of c-*myc*. While *CDKN2A* downregulation was not sufficient to make cells tumorigenic, the ability to induce tumors in nude mice correlated with p53 inactivation and c-*myc* overexpression. During further culture propagation, cen3tel cells showed a shorter latency in inducing tumors, suggesting that they had acquired increased tumorigenicity and could be a useful tool for obtaining further information on molecular changes associated with tumor progression [23].

In this study we found that, upon neoplastic transformation, cen3tel cells increased their migratory and invasive capacity by adopting a protease-independent/ROCK-dependent mechanism of invasion. We show here that Rnd3 (also known as RhoE), a cellular inhibitor of ROCK-I [25], plays a relevant role in regulating invasion and metastasis formation of sarcoma cells.

## Materials and Methods

### Ethics Statement

Procedures involving animals and their care were conducted in conformity with the institutional guidelines that are in compliance with national (Decreto Legge No. 116, Gazzetta Ufficiale, Suppl. 40, Feb. 18, 1992; Circolare No. 8, Gazzetta Ufficiale, July, 1994) and international laws and policies (European Economic Community Council Directive 86/609, Official Journal Legislation 358. 1, Dec. 12, 1987; Guide for the Care and Use of Laboratory Animals, United States National Research Council, 1996). The study was reviewed and approved by the IRFMN Animal Care and Use Committee (IACUC), which includes “ad hoc” members for ethical issues. Aproval ID Frap1.

### The cen3tel cellular system

The cen3tel telomerase immortalized cell line was obtained from primary cen3 fibroblasts, derived from a centenarian, by infection with an hTERT-containing retrovirus [22]. Cen3tel cells were used at different steps of propagation reflecting different phases of transformation [23]. In particular, we used cen3tel cells at five phases of propagation (up to around population doubling (PD) 1000): early cen3tel cells, these cells are at the initial passages after infection with the hTERT containing retrovirus (between PDs 34 and 45) and show a behaviour similar to that of primary cen3 fibroblasts; mid cen3tel cells (around PD 100), these cells are at an early phase of transformation, they are able to grow in the absence of solid support, but are not tumorigenic in nude mice; tumorigenic cen3tel cells, which induce tumors when inoculated subcutaneously into nude mice, were further subdivided in three groups, according to the time required for tumor formation, which decreases at increasing PDs (see first section of the Results), tumorigenic cells of phase I (around PD 160), phase II (around PD 600) and phase III (around PD 1000).

### Cell culture, transfection and plasmid

Primary and immortalized cells were grown in Dulbecco's modified Eagle's Medium (DMEM, Celbio) supplemented with 10% fetal bovine serum (Lonza), 2 mM glutammine, and 1% non-essential amino acids (Euroclone), 0.1 mg/ml penicillin (Euroclone), 100 U/ml streptomycin (Euroclone) at 37°C in an atmosphere containing 5% CO_2_. To analyze cellular morphology and organization of the actin cytoskeleton (see section “Immunofluorescence”) in a 3D environment, cells were plated on top of Matrigel (8 mg/ml) (BD Biosciences) in 8 chamber polystyrene vessels (BD Transduction Laboratories Biosciences Falcon) and incubated at 37°C with 5% CO_2_ in complete DMEM to allow Matrigel invasion. Cells in Matrigel were treated with 10 µM Y27632 (Calbiochem) for 6 hours. Cell morphology was observed using an optical microscope Olympus IX71 equipped with a 4x objective (NA 0.13). Images were taken with a digital camera Cool SNAP_ES_ (Photometrics) using the MetaMorph software.

Cells were transfected with the linearized plasmids EGFP-C1 (Clontech) or EGFP-C1-Rnd3 (kindly provided by Dr. Pierre Roux, CRBM-CNRS FRE2593, France) using Lipofectamine 2000 (Invitrogen) according to the manufacturer's instructions. Transfected cells were selected and expanded in complete medium containing 0.5 mg/ml G418 (Invitrogen).

### Motility and Invasion Assays

Cell motility and invasiveness were assayed using modified Boyden chambers with polycarbonate PVP-free Nucleopore filters (8 µm pore size) [26]. Supernatant of NIH-3T3 cells was used as a reference attractant and was added to the lower compartment of the Boyden chamber. For motility, filters were coated with 0.1% gelatin. For invasion, filters were coated with a thick layer of the reconstituted basement membrane Matrigel (0.5 mg/ml; Becton Dickinson). Cells were detached, washed in DMEM containing 0.1% BSA, resuspended in the same medium at a concentration of 5×10^5^/ml, and added to the upper compartment of the chamber. When indicated, the ROCK inhibitor Y27632 (Calbiochem) or the MMP inhibitor Ro 28–2653 (kindly provided by H.W. Krell, Roche Diagnostics Gmbh, Penzberg, Germany) was added to the cells 1 hour before the assay and left throughout the assay. After 4 hours (motility) or 6 hours (invasion), filters were stained with Diff-Quik (Baxter), and migrated cells in 10 high-power fields were counted. We choose these times of analysis to avoid that possible proliferation differences between cell lines could affect the results. Results are expressed as the number of migrated or invaded cells or as the percentage of control invasion (inhibitor treated or transfected cells). Statistical differences between groups were evaluated by ANOVA followed by Bonferroni *post hoc* test.

### Immunofluorescence

Cells were seeded at different density in 12 well plates (Corning) containing 19 mm diameter coverslips and incubated at 37°C for 24 hours. Filamentous actin (F-actin) was stained with phalloidin-TRITC (P1951, Sigma-Aldrich) diluted 1∶300 and nuclei with 2 µg/ml 4′,6-diamidino-2-phenylindole (DAPI) in PBS for 10 min. The phosphorylated form of myosin II regulatory light chain (p-MLC) was stained using the anti-pS19-MLC primary antibody (#3671, Cell Signaling) diluted 1∶50 and an anti-rabbit secondary antibody conjugated with FITC (Jackson Immunoresearch), after an overnight block in 0.3% BSA in PBS at 4°C. To detect F-actin in cells in Matrigel matrix, cells were fixed for 30 minutes with 4% paraformaldehyde-0.25% glutaraldheyde in PBS on ice and then treated for 30 minutes with 0.2% Triton-X 100 in PBS. Cells were then incubated with phalloidin-TRITC (P1951, Sigma-Aldrich) diluted 1∶1000 and with 0.2 µg/ml DAPI for 3 hours. Slides were analyzed with the Leica TCS SP2 confocal laser microscope using a 40x objective (NA 1.32). Figures were assembled using Adobe Photoshop and Adobe Illustrator.

### Western blot analysis

Whole-cell lysates were prepared using the RIPA buffer (50 mM Tris-HCl pH 8, 150 mM NaCl, 1% Nonidet P40, 0.1% SDS, 0.1% DOC, 1x protease inhibitor cocktail (Roche) and 0.2% Na_3_VO_4_) or the Laemmli buffer (60 mM Tris-HCl, pH 6.8, 10% glycerol, 5% β-mercaptoethanol, 2% SDS, 0.02% bromophenol blue). The anti-Rnd3 antibody [R 6153, clone 4 (Sigma-Aldrich), dilution 1∶500] was used on extracts prepared with the RIPA buffer. The anti-pS19-MLC (1∶500) was used on extracts prepared with the Laemli buffer and blotted onto PVDF membranes (Biorads). The anti-γ-tubulin antibody (T6557, clone GTU-88, Sigma-Aldrich) was used as control for protein loading. Secondary Horseradish Peroxidase conjugated antibodies were from Jackson ImmunoResearch (anti-mouse IgG 115-035-146, anti-rabbit IgG 111-035-003). Chemiluminescent assays (Pierce) were used to detect the secondary antibody signal.

### RNA extraction and microarray analysis

Total RNA was extracted using the Trizol reagent (Invitrogen) from actively dividing primary fibroblasts (PD 15) and from cen3tel cells representing the five phases of propagation (PD 37, 97, 167, 618, 1032 and 1042; cells at PDs 1032 and 1042 both represent cells of tumorigenic phase III). Microarray probe preparation, hybridization on Agilent Whole Genome 44k oligo microarrays and scanning were carried out as previously described [27]. Images were analyzed using Feature Extraction software (Agilent Technologies) version 8.1. Output files were then treated with the Limma (linear models for microarray data) package [28] available within Bioconductor (http://www.bioconductor.org/). Linear models were fitted to the normalised data to compare cen3tel cells at each population doubling with control cen3 fibroblasts. The empirical Bayes method was used to compute the moderated t-statistic and F-statistic [29]. P-values were adjusted for multiple testing by the Benjamini-Hochberg approach [30]. To select differently expressed transcripts, a cut-off of 0.01 was applied to the adjusted p-value. Microarray data have been deposited in the NCBI database GEO (Gene Expression Omnibus), accession number GSE157442.

### Animal models

The *in vivo* tumorigenic potential of cen3tel cells was evaluated by injecting 10^7^ cells in the right flank of female athymic nude mice or female SCID mice (Harlan Italy, Bresso, Italy). Mice were monitored two/three times a week to assess tumor appearance and growth. To investigate the metastatic ability of cen3tel cells, 2×10^6^ cells were injected i.v. in nude or SCID mice. Mice were monitored daily and sacrificed at the appearance of distress symptoms. Animals were autopsied to evaluate metastases. To establish the capacity of primary tumors to give spontaneous metastases, tumor masses obtained after s.c. injection of cen3tel cells were surgically removed under isoflorane anesthesia and the mice were kept alive until the appearance of distress symptoms. After sacrifice, animals were autopsied to evaluate metastases. For counting metastatic foci, tissues were collected and stored in BOUIN. For histopathologic examination, tumor masses and animal parenchymal organs were fixed in 10% buffered formalin, and paraffin embedded. 4-µm sections were cut and stained with hematoxylin and eosin.

## Results

### *In vivo* tumorigenicity and metastatic potential of cen3tel cells at different stages of propagation

**Figure 1:**
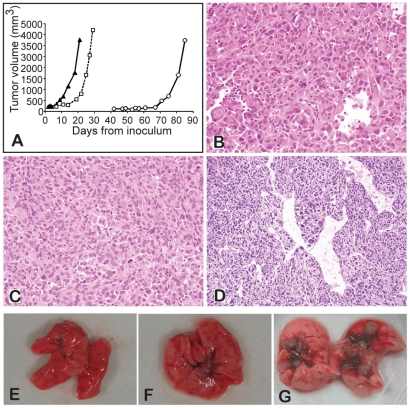
Tumor induction by cen3tel cells. A) Growth curves of tumors induced by cen3tel cells at PD 165 (empty circles), PD 616 (empty squares) and PD 902 (black triangles). B–D) Histological analysis (hematoxylin and eosin staining) of tumors induced by cen3tel cells at PD 165 (B), PD 616 (C) and PD 977 (D). E–G). Lungs from mice intravenously injected with tumorigenic cen3tel cells of phase I (E), phase II (F) or phase III (G), only in (G) are metastases visible.

Cen3tel cells at different PDs were inoculated subcutaneously in immunocompromised mice. Analysis of cen3tel cells at tumorigenic phase I and phase II (respectively around PD 160 and PD 600, see Material and Methods for the description of the cellular system) confirmed our previous results [23], with tumors becoming detectable with latencies of about one month and eight days, respectively (Fig. 1A). Conversely, phase III tumorigenic cen3tel cells (PD 900–1020) generated tumors already evident two days after inoculation (Fig. 1A). Histological analysis revealed that the tumors developed by cen3tel cells at the first and second tumorigenic phases were pleomorphic sarcomas (Fig. 1B and C), those developed by phase III cen3tel cells showed a hemangiopericytoma-like vascular pattern, similar to human poorly differentiated, round-cell synovial sarcoma (Fig. 1D). Cen3tel cells of tumorigenic phase III recapitulated the histological features of synovial sarcomas even in the absence of the SYT-SSX fusion transcript (data not shown), which is diagnostic of this sarcoma subtype [31], suggesting the involvement of other genetic or epigenetic mechanisms, possibly associated with the still poorly understood SYT-SSX downstream targets.

Cen3tel cells at different PDs were also injected intravenously to test whether they were able to give metastases. Both cen3tel cells at tumorigenic phases I and II did not induce metastases (in 3 mice each, Fig. 1E and F). In contrast, in the lung of 10 out of 10 mice injected with cen3tel cells around PD 1000, a high number of metastases (always more than 50 metastatic foci *per* lung) were observed 4 weeks after injection (Figure 1G).

### Tumorigenic cen3tel cells are more motile and invasive than non-tumorigenic cells

**Figure 2:**
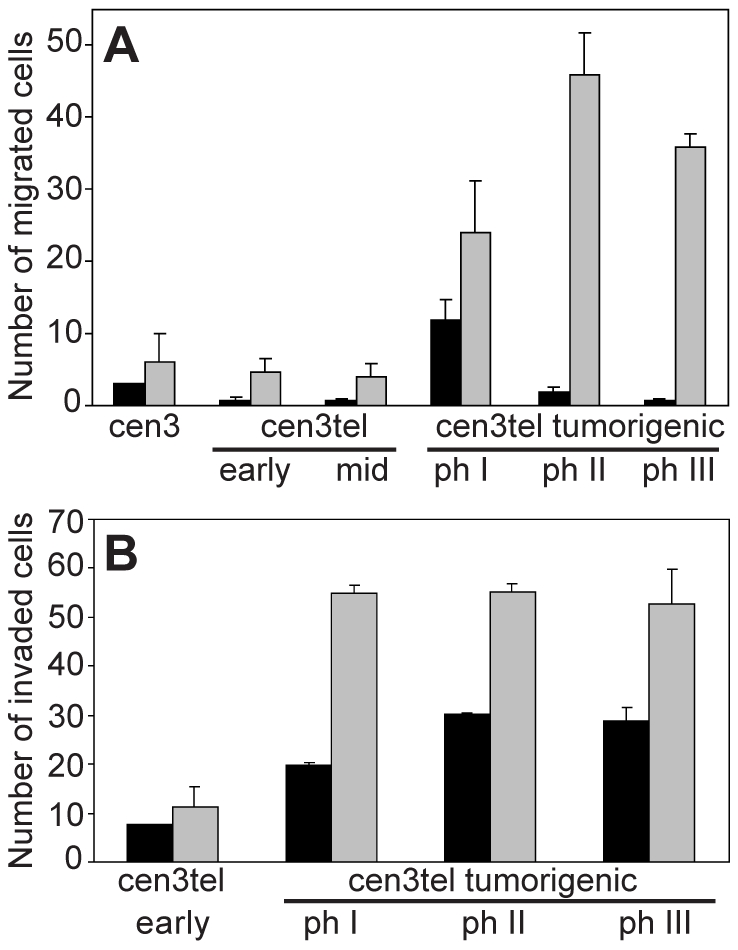
Motility and invasiveness of cen3tel cells at different PDs. A) Motility and (B) invasiveness were assessed in Boyden chambers, in the absence (spontaneous migration, black columns) or in the presence (grey columns) of NIH-3T3 supernatant, used as chemoattractant. To assess invasion, the membrane of the Boyden chambers was covered with a thick layer of Matrigel. The numbers of migrated cells, mean and SE from 2–6 independent experiments are shown. Stimulated migration and invasion of tumorigenic cen3tel cells were significantly higher (respectively p<0.01 and p<0.05 for migration and invasion) than that of cen3 fibroblasts or non-tumorigenic cen3tel cells.

To study motility and invasiveness in cen3tel cells at different PDs, we used Boyden chambers with filters coated either with gelatin or with a thick layer of the reconstituted basement membrane Matrigel. The number of cells able to migrate through the porous membrane coated by gelatin was considered a measure of the migratory capacity, and the number of cells passing through Matrigel a measure of the invasive capacity. As illustrated in Figure 2A, which shows both spontaneous motility (black columns) and motility in response to a chemoattractant (grey columns), parental cen3 fibroblasts, as well as early and mid cen3tel cells, had a very limited migratory potential; in contrast, tumorigenic cen3tel cells were clearly able to migrate through the porous membrane. The same holds true for the ability to invade Matrigel (Fig. 2B). Interestingly, invasiveness was also high in the absence of the chemoattractant. Thus, cen3tel neoplastic transformation is associated with increased migratory and invasive potential. In the *in vitro* experiments, we could see no clear difference in the migratory or invasive potential between the cells in the three phases of tumorigenicity. In particular, in cen3tel cells around PD 1000, there was no clear increase in *in vitro* invasion, that could be related to the acquisition of the metastatic ability.

### Tumorigenic cen3tel cells show actin cortical rings and a spherical morphology in a 3D environment

**Figure 3:**
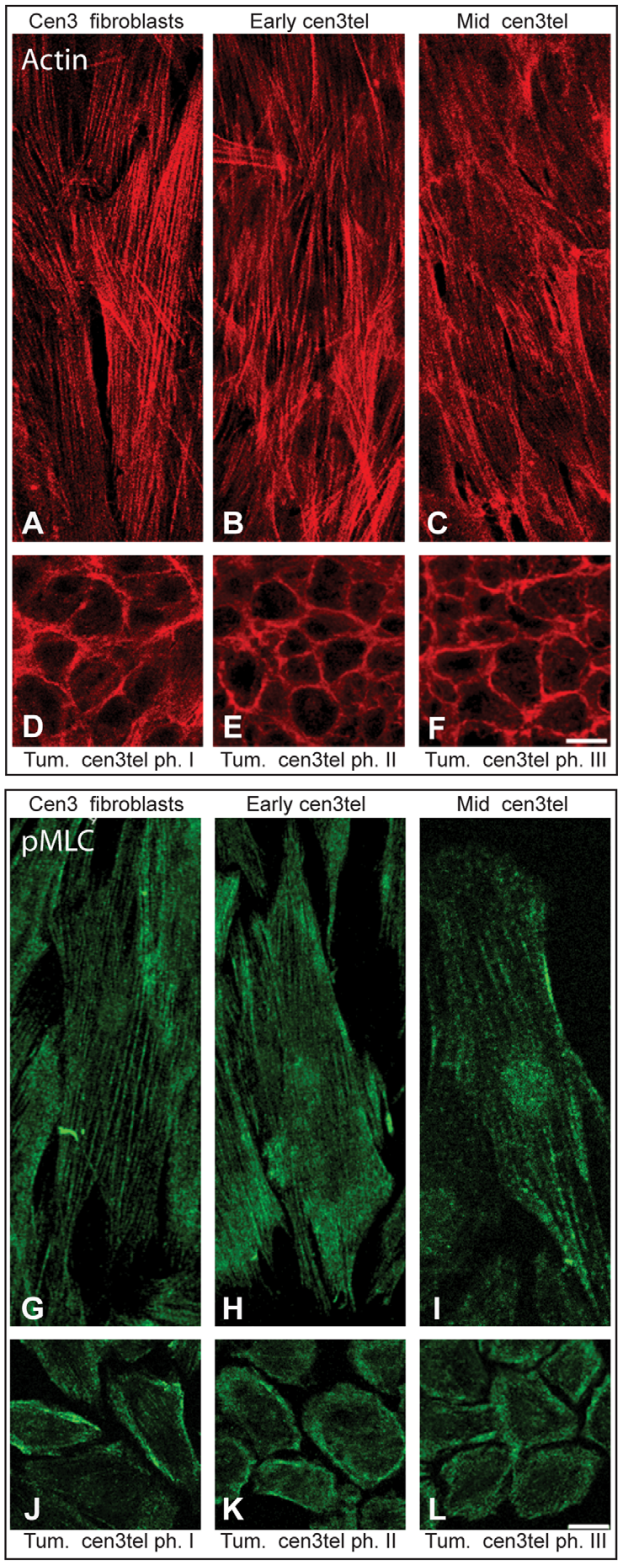
Actin and pMLC cellular distribution in cen3 primary fibroblasts and cen3tel cells. To detect actin, cells were seeded on a coverslip and incubated with TRITC-labelled phalloidin (A–F, red signal), which binds to F-actin. Tumorigenic cells show a polygonal shape compared to non-tumorigenic ones and show actin cortical rings. To detect pMLC, indirect immunofluorescence was done with an anti-pMLC primary antibody and a FITC conjugated secondary antibody (G–L, green signal). In tumorigenic cells, PML is mainly distributed along the inner membrane. (Images were taken with a confocal microscope, 40x objective, bars 25 µm; single confocal sections are shown).

Cen3tel cells routinely grown on plastic showed a clear change in morphology during culture propagation. Cells switched from a typical elongated fibroblastic shape, distinctive of cells up to around PD 100, to a polygonal shape characteristic of cells that had become neoplastically transformed [23]. To test whether neoplastic transformation and the change in cell morphology were associated with changes in the actin cytoskeleton, we analyzed the organization of the actin filaments in cells at different stages of propagation. We stained F-actin using its interacting molecule phalloidin, conjugated to a fluorochrome. A clear difference in F-actin organization was observed between tumorigenic and non-tumorigenic cen3tel cells (Fig. 3A–F). As expected, F-actin was organized in stress fibers in primary cen3 fibroblasts (Fig. 3A) and the same organization was also observed in early and mid cen3tel cells (Fig. 3B, C). In tumorigenic cen3tel cells (Fig. 3D–F), phalloidin staining clearly highlighted the change in morphology with cortical rings formed along the inner cell periphery.

**Figure 4:**
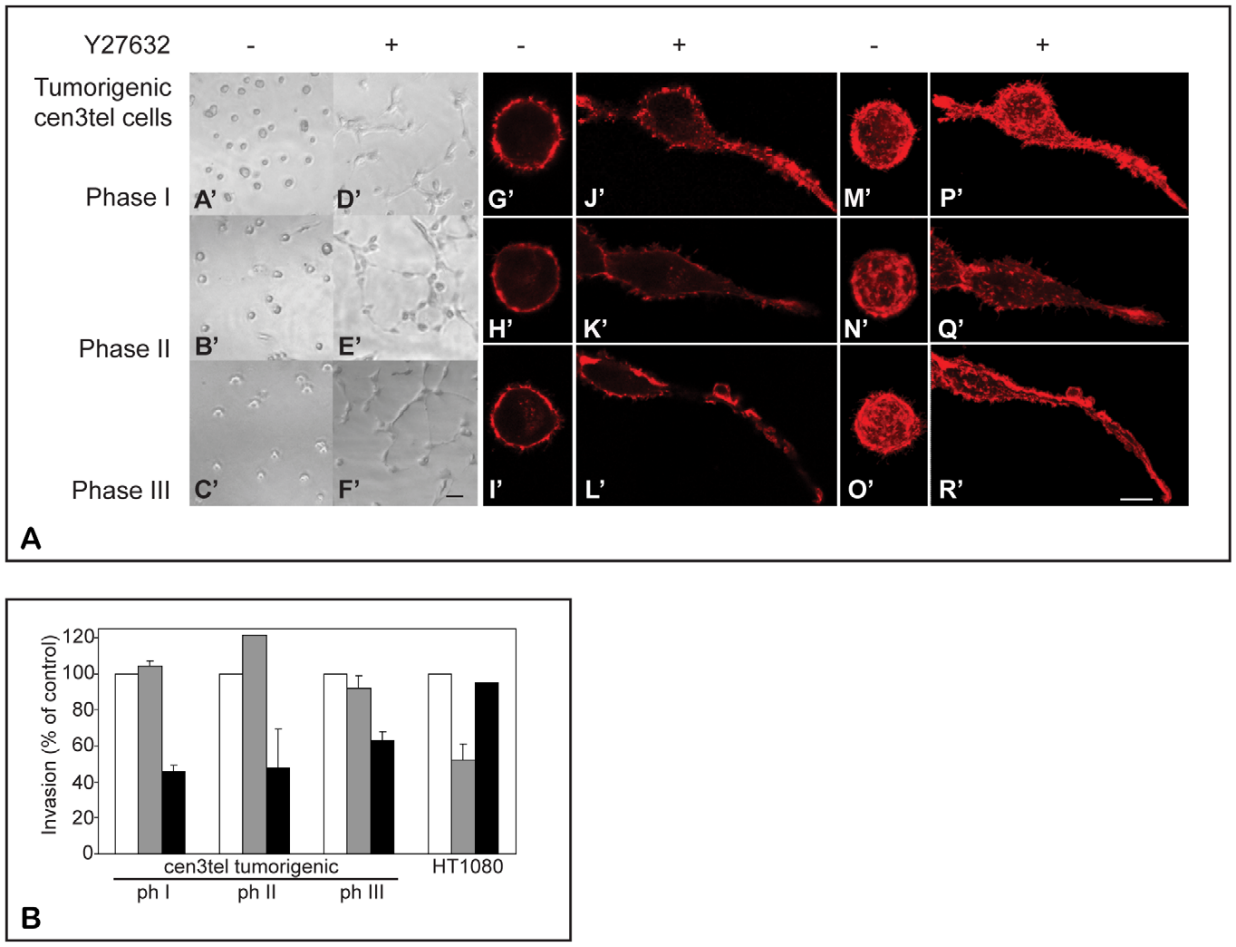
Morphology in 3D and control of invasion in tumorigenic cen3tel cells. A) Morphology and F-actin organization of tumorigenic cen3tel cells embedded in Matrigel matrix and effect of the ROCK inhibitor Y27632. Left panels A'–F': phase contrast images (10x objective, bar 50 µm). Right panels: direct immunofluorescence with TRITC-labelled phalloidin. Iimages were taken using a confocal microscope (40x objective, bar 10 µm); G'–L': single confocal sections, M'–R': average of multiple confocal sections. 3D reconstruction of the image in panel O' is showed in Supplementary Video 1. B) Effect of the ROCK inhibitor Y27632 and of the MMP inhibitor Ro 28–2653 on invasion of cen3tel cells at different PDs and of the fibrosarcoma cell line HT1080. Invasion was measured using Boyden chambers. Black columns: Y27632 (10 µM); grey columns: Ro 28–2653 (0.1 µM). The percentages of invasion of control cells (in the absence of inhibitors, white columns) are shown, mean and SE from 2–4 independent experiments.

Together with the change in actin organization, there was a parallel re-distribution of p-MLC (Fig. 3G–L). In fact, while in non-tumorigenic cells, the phosphorylated form of myosin II was distributed in fibers along the cells (Fig. 4G–I), in tumorigenic cells it mainly formed a ring around the inner cell periphery (Fig. 4J–L).

The change in cytoskeletal organization observed in tumorigenic cen3tel cells suggests that the transformed cells could have switched to an amoeboid-type movement. Cells migrating through this movement have a characteristic rounded shape when grown in a deformable gel like Matrigel and show cortical actin rings with membrane blebbings [6]. To analyze cen3tel tumorigenic cell morphology and actin organization in 3D, we plated them on the top of Matrigel and allowed them to invade the gel. After 24 hours, cells were first analyzed by phase contrast microscope (Fig. 4A, A'–C') and then fixed and stained with phalloidin conjugated with TRITC (Fig. 4A, G'–I', M'–O'). Tumorigenic cells had a rounded morphology (A'–C'), with F–actin cortical rings (G'–I', M'–O') (see also Movie S1). It is worth noticing that the rounded morphology was also observed in cells of the sarcoma-like tumor masses generated by cen3tel cells in animal models.

### ROCK inhibition leads to an elongated morphology and reduces cen3tel cell invasion

Rounded cell morphology and cortical actin rings depend on the activity of the Rho-effector kinase ROCK, which acts by increasing myosin-II-mediated actin filament stabilization and contraction [32]. Inhibition of ROCK causes the loss of the spherical cellular morphology [10]. To test whether this was the case in tumorigenic cen3tel cells, we exposed cells that had invaded Matrigel to the ROCK chemical inhibitor Y27632. Treatment with the inhibitor produced a more elongated morphology (Fig. 4A, D'–F') and altered the actin cortical rings (Fig. 4A, J'–L', P'–R'), indicating that ROCK activity is involved in the change of shape of tumorigenic cen3tel cells.

It is known that the invasiveness of rounded tumor cells depends on ROCK activity, while it is independent of the activity of matrix proteases [10], [19]. Global gene expression profiling of cen3tel cells at different PDs showed a decrease in the expression of different types of extra-cellular matrix proteases during cen3tel propagation *in vitro*, particularly of several matrix-metalloproteases (MMPs) (Table 1), and zymographic analysis showed a decrease in the activity of MMP2 and MMP9 in tumorigenic cen3tel cells (data not shown), suggesting that increased motility in late cen3tel cells was not strictly dependent on pericellular matrix proteolysis.

**Table 1:** Expression of different matrix metalloproteinase (MMP) genes in cen3tel cells at different PDs, relative to parental cen3 fibroblasts*.

Gene	Accession Number	Cen3telPD 37	Cen3telPD 97	Cen3telPD167	Cen3telPD 618	§Cen3telPD 1034	§Cen3telPD 1042
MMP1	NM_002421	−1.2	−3.8	−4.3	−4.1	−4.4	−4.5
MMP2	NM_004530	+0.5	−2.2	−3.0	−2.1	−3.8	−4.1
MMP3	NM_002422	−1.2	−2.9	−3.5	−2.9	−2.8	−2.9
MMP7	NM_002423	−0.4	−0.7	−0.8	−0.7	−1.6	−1.5
MMP9	NM_004994	−0.6	−0.5	−0.6	−0.6	−1.1	−1.0
MMP10	NM_002425	−2.2	−1.8	−2.9	−2.8	−3.5	−3.6
MMP11	NM_005940	+1.3	−1.9	−1.9	−2.1	−2.6	−2.1
MMP12	NM_002426	−4.0	−3.9	−5.3	−5.4	−5.9	−6.0
MMP14	NM_004995	+0.5	−0.7	−2.3	−2.7	−1.9	−1.9
MMP19	NM_022791	−0.6	+0.1	−1.7	−1.7	−2.1	−2.0
MMP27	NM_022122	−0.2	−1.9	−2.0	−1.9	−2.5	−2.6
MMP8	NM_002424	+0.3	−0.5	−0.7	−0.5	−1.2	−0.9
MMP25	NM_022718	−0.3	+0.1	+0.4	+0.6	+0.5	+0.6

*The values are the log2 of the ratio.

§ Cen3tel 1034 and cen3tel 1042 represent cells of tumorigenic phase III.

To test the roles of ROCK and metalloproteases on cen3tel cell invasiveness, we analyzed the invasive ability of cen3tel cells at the three stages of tumorigenesis in the presence of the ROCK inhibitor Y27632 or in the presence of the metalloprotease inhibitor Ro 28–2653. As shown in Fig. 4B, Y27632 reduced the invasiveness of cen3tel cells at all three different stages (black columns), while the metalloprotease inhibitor did not affect it (grey columns). The opposite result was observed in HT1080 cells, which use mesenchymal invasion (Fig. 4B). These results indicate that tumorigenic cen3tel cells acquired a protease-independent type of invasion, which requires the activity of ROCK kinase.

### Rnd3 expression inversely correlates with the invasive and metastatic properties of tumorigenic cen3tel cell

**Figure 5:**
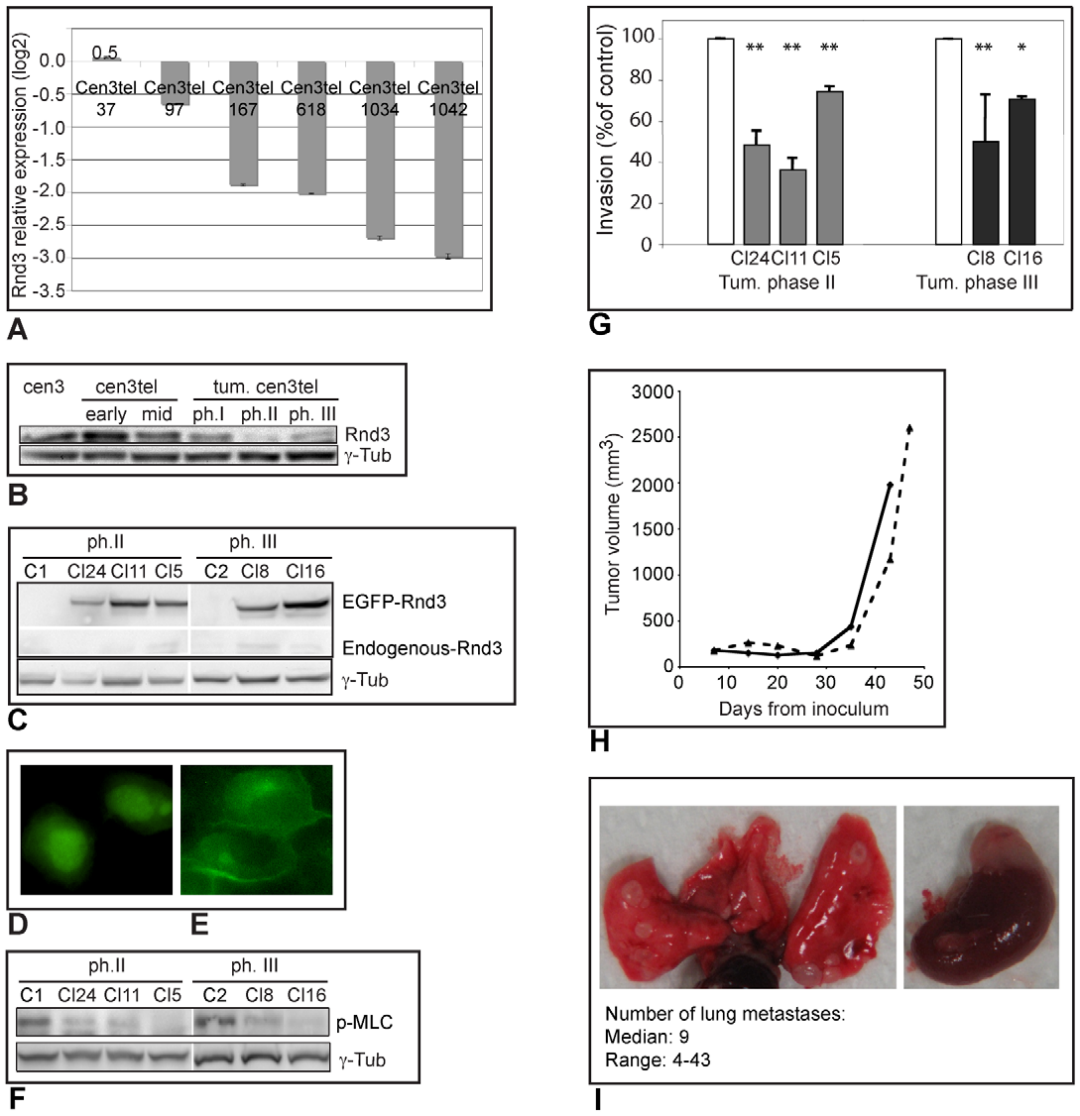
*Rnd3* expression in cen3tel cells and its effect on their invasive and metastatic capacity. A) Results of microarray analysis. *Rnd3* expression in cen3tel cells is indicated relative to cen3 primary fibroblasts. The values are the average of the results for ten spots corresponding to the same probe for *Rnd3* (bar: standard error); cen3tel at PD 1034 and at PD 1042 both represent cells of tumorigenic phase III. B) Western blotting analysis of Rnd3 expression in primary cen3 fibroblasts and cen3tel at different stages of transformation. γ-tubulin was used as loading control. C) Western blotting analysis of recombinant EGFP-Rnd3 expression in transfected clones. C1 and C2 are mock-transfected clones from phase II and III tumorigenic cen3tel cells, respectively (the endogenous protein and the recombinant one were detected on the same membrane with the anti-Rnd3 antibody, but part of the space between the two signals has been eliminated to make the figure smaller). γ-tubulin was used as loading control. D) Subcellular localization of EGFP (yellow signal) or E) of the EGFP-Rnd3 fusion protein in cells stably transfected with either the empty vector or the Rnd3 expression vector. The exogenous Rnd3 protein is localized around the plasma membrane and in the cytoplasm where there is a perinuclear accumulation. Images were taken using an optical microscope (60x objective). F) Western blot analysis of the levels of pMLC in Rnd3-expressing and mock-transfected clones (C1 and C2). γ-tubulin was used as loading control. G) Invasion of clones from phase II (grey bars) and III (black bars) tumorigenic cen3tel cells transfected with the Rnd3expression vector. Invasion is expressed as the percentage of invasion in cells transfected with the empty vector (white bars). The average of the results from at least two independent experiments is shown (*: p<0.05; **: p<0.001). H) Growth curves of tumors obtained in SCID mice after subcutaneous inoculation of phase III tumorigenic cen3tel cells, either mock-transfected (continuous line) or stably transfected with the Rnd3 expression vector (dashed line). I) Lungs (left) and adrenal gland (right) from SCID mice six weeks after intravenous inoculation of the mock-transfected phase III tumorigenic cen3tel cells; metastases are visible on both organs. In the box, the median number of metastases *per* animal and the range are reported.

By microarray analysis and western blotting we did not observe any evident change in ROCK or RhoA expression in tumorigenic cen3tel cells (data not shown); however, microarray and western blot analyses revealed that the cellular inhibitor of ROCK-I, Rnd3 (Riento et al. 2003), was expressed at lower levels in tumorigenic cen3tel cells compared to non-tumorigenic ones (Fig. 5A, B), suggesting that Rnd3 downregulation might play a role in the transition to the ROCK dependent mode of invasion.

To test whether Rnd3 levels affected tumorigenic cen3tel cell invasiveness, we transfected phase II and phase III tumorigenic cells with an EGFP-Rnd3-expression vector and isolated clones with exogenous Rnd3 expression, three from phase II tumorigenic cen3tel cells and two from phase III cells (Fig. 5C). Compared to mock-transfected cells, in which the EGFP signal was, as expected, both nuclear and cytoplasmic (Fig. 5D), in the cells transfected with the recombinant vector, the EGFP-Rnd3 fusion protein showed the same subcellular localization described for the endogenous protein [33]: it was present in the cytoplasm, at the plasma membrane and showed a perinuclear accumulation suggestive of a localization in the Golgi apparatus (Fig. 5E). Rnd3 expressing clones showed proliferation rates similar to that of parental and mock transfected cells (data not shown).

Rnd3 overexpression induces actin fiber disassembly in several cell types [34]. In the clones stably expressing Rnd3, we found no major changes in cellular morphology compared to parental cells, or actin depolymerization (not shown). This might be related to the level of expression of the protein in the clones, which may not be high enough and, above all, to its stable expression. In fact, it has been shown that in mouse 3T3 fibroblasts, the strong effects induced by acute Rnd3 expression on the cytoskeleton are only transient and disappear within hours, even though the levels of the protein are still high [35]. In the Rnd3 expressing clones we did find lower level of p-MLC than in parental and mock-transfected cells, which could be due to an Rnd3-induced reduction in ROCK kinase activity (Fig. 5F).

We analyzed invasion in the five Rnd3 expressing clones and, in all of them, it was lower than in mock transfected cells (Fig. 5G), indicating that Rnd3 expression can modulate invasion of neoplastically transformed human fibroblasts.

We then investigated whether exogenous Rnd3 expression reduced the *in vivo* metastatic potential of phase III tumorigenic cen3tel cells. We analyzed the metastatic potential of Rnd3 clone 16, because of the greater stability of Rnd3 expression in this clone compared to that in clone 8 (not shown). We first inoculated mock-transfected control cells and Rnd3 clone16 cells subcutaneously in nude mice, to verify their tumorigenic potential. Cells from both transfected populations developed tumors; however, after about 10 days, when tumors were around 250 mm^3^, the tumors were rejected, probably because of an immune response to EGFP expressing cells [36]. To avoid rejection, we inoculated the cells into SCID (Severe Combined Immuno-Deficiency) mice, which are deficient in T and B cells. In these mice, both cell lines developed tumors, which showed similar growth rates, indicating that Rnd3 does not affect subcutaneous tumor growth (Fig. 5H). To analyse metastasis formation, cells were injected intravenously in SCID mice. Mice were sacrificed after about six weeks; metastatic masses were detected in the lungs (from 4 to 43 metastases/mouse with a median number of 9) and in the adrenal glands of the six mice inoculated with mock-transfected cells (Fig. 5I), while they were not observed in any of the eight mice inoculated with Rnd3 clone 16 cells.

Taken together, these results indicate a role for Rnd3 downregulation in promoting invasion and metastasis formation by mesenchymal tumor cells.

## Discussion

The cen3tel cellular system [22], [23], derived from human telomerase immortalized fibroblasts, has allowed us to follow the stepwise process of cellular transformation, from normal fibroblasts up to tumorigenic cells with metastatic potential. In this paper, we focused on the study of molecular and biological features associated with changes in invasiveness during the progression from normal to tumorigenic and metastatic fibroblasts. During transformation, cen3tel cells increased their migratory and invasive potential and gradually acquired the ability to induce metastases in immunocompromised mice. We show here that the increased invasiveness does not depend on metalloprotease activity, as expected for cells of mesenchymal origin, but on the activity of the ROCK kinase; in addition, we show that Rnd3 negatively regulates invasion and metastasis formation.

Although ROCK-dependent invasion was first described in carcinoma and melanoma cell lines [10], evidence has been recently reported that cells of mesenchymal origin can adopt a protease-independent amoeboid type of movement, making ROCK a possible therapeutic target for sarcomas [37]. In mouse 3T3 fibroblasts a switch to amoeboid movement was observed upon p53 inactivation [14], while up-regulation of the Rho/ROCK signalling was found in highly metastatic rat sarcoma cells, together with the loss of MMP2 activity and an increased generation of protrusive forces, typical of the amoeboid movement [21]. Moreover, it has been shown that microtubule destabilization through stathmin overexpression can also lead to acquisition of amoeboid movement in sarcoma cells and that the tumor suppressor protein p27^kip1^ is an important factor for the control of cellular morphology and motility of transformed fibroblasts by regulating microtubule stability [38], [39].

In cen3tel cells, neoplastic transformation was associated with an increasing invasive capacity, linked to a change in cell morphology from elongated to rounded, in the organization of the actin cytoskeleton from stress fibers to cortical rings, and in the subcellular distribution of p-MLC. Chemical inhibition of ROCK led to a reversal of the rounded morphology and to a decrease in the invasive capacity. Unlike in HT1080 fibrosarcoma cells, we found that invasion of tumorigenic cen3tel cells was not reduced upon treatment with a matrix protease inhibitor, indicating that cells of mesenchymal origin can spontaneously undergo a transition towards amoeboid movement during transformation.

At the molecular level, this switch could be linked to two main changes: the decrease in the expression of matrix protease genes and the reduced expression of the Rho GTP-binding protein Rnd3. Experimental inhibition of pericellular proteolysis can induce a transition from mesenchymal to amoeboid movement [19]; in our cellular system, spontaneous downregulation of matrix protease genes, through a mechanism still to be clarified, might have contributed to the switch in the type of movement.

Rnd3, together with Rnd1 and Rnd2, belongs to the Rnd family of small GTP-binding proteins always bound to GTP, which is involved in the control of cytoskeletal organization [25]. Microarray analysis did not show any significant change in the expression of *Rnd1* and *Rnd2* during malignant transformation of cen3tel cells. Rnd3 is an inhibitor of ROCK-I [32], [40] and evidence has been reported that it can play a role in controlling morphology and invasion of rounded tumor cells. Pinner and Sahai [41] showed that a fine balance between PDK1 and Rnd3 expression is required for the amoeboid movement of melanoma cells, because the two proteins have opposing roles in controlling ROCK-I activity. PDK1 drives ROCK-I to the plasma membrane where it stimulates actomyosin phosphorylation and contraction, while Rnd3 can bind to ROCK-I and inhibit its activity, reducing motility. Gadea et al. [14] showed that overexpression of Rnd3 in mouse embryonic fibroblasts in which amoeboid migration had been induced by *p53* knockdown led to reduced invasion. Our observation that ectopic Rnd3 expression in phase II and III tumorigenic cen3tel cells reduced their invasive capacity points to Rnd3 as a possible regulator of invasion of cells of mesenchymal origin. In addition, the low levels of myosin phosphorylation in Rnd3 transfected clones is in agreement with the hypothesis that Rnd3 hampers amoeboid movement by inhibiting ROCK kinase activity. Finally, the low metastatic potential observed in phase III tumorigenic cen3tel cells that ectopically express Rnd3 suggests that Rnd3 exerts its effect also *in vivo* and that its downregulation can have a pro-metastatic effect.

*Rnd3* shows reduced expression in prostate cancer and seems to have a protective role against breast cancer [25]; however, increased *Rnd3* expression has been found in other epithelial tumors and in metastatic melanoma cells [42], suggesting that the genetic background of cancer cells can influence the role of *Rnd3* in trasformation. Very little is known about *Rnd3* expression in human sarcomas. Our results indicate that reduced *Rnd3* expression in tumor cells of mesenchymal origin can be linked to the acquisition of a ROCK-dependent mode of invasion and can increase the metastatic potential. Thus, analysis of *Rnd3* expression in human sarcomas might give information on the mechanism of invasion adopted by the tumor cells and help orientate the therapeutic strategy [37].

It is worth noticing that the increase in the invasive potential and *Rnd3* downregulation occur when cen3tel cells are tumorigenic but not yet metastatic. This suggests that the acquisition of invasive ability by tumor cells, although necessary, is not sufficient to give them full metastatic ability. Recent studies [43]–[47] suggest that even normal cells can disseminate and dissemination from primary tumors can actually precede the ability to form metastases; additional genetic or epigenetic changes are then required for the subsequent production of metastases. It is possible that cen3tel cells progressively acquired different and complementary malignant properties besides invasiveness that ultimately led to a fully metastatic cell population. In agreement with this, preliminary findings indicate a progressive increase in the expression of pro-angiogenic factors (such as VEGF and FGF2) and a concomitant decrease of angiogenesis-inhibitory factors (particularly TSP-1 and TSP-2) during cen3tel malignant progression, pointing to angiogenesis as a candidate pro-metastatic feature acquired by late-phase metastatic cen3tel cells.

In conclusion, the results presented here do suggest that sarcoma cells can adopt a protease-independent/ROCK-dependent mechanism of invasion and indicate that the *Rnd3* gene can play a role in regulating the invasiveness and metastatic capacity of mesenchymal tumor cells.

## Supporting Information

Movie S1 Tumorigenic cen3tel cells have a rounded morphology. 3D reconstruction of a tumorigenic phase III cen3tel cell in Matrigel in which F-actin was highlighted using TRITC-labelled phalloidin and the nucleus by staining with DAPI. Images of different focal planes were taken with a confocal microscope (objective 40x).
(0.05 MB MOV)
